# Catalytic Ammonia Synthesis Mediated by Molybdenum Complexes with PN^3^P Pincer Ligands: Influence of P/N Substituents and Molecular Mechanism

**DOI:** 10.3390/molecules27227843

**Published:** 2022-11-14

**Authors:** Katja Bedbur, Nadja Stucke, Lina Liehrs, Jan Krahmer, Felix Tuczek

**Affiliations:** Institut für Anorganische Chemie, Max-Eyth-Straße 2, 24118 Kiel, Germany

**Keywords:** small molecule activation, dinitrogen activation, ligand design, catalysis, ammonia

## Abstract

Three molybdenum trihalogenido complexes supported by different PN^3^P pincer ligands were synthesized and investigated regarding their activity towards catalytic N_2_-to-NH_3_ conversion. The highest yields were obtained with the H-PN^3^P^tBu^ ligand. The corresponding Mo(V)-nitrido complex also shows good catalytic activity. Experiments regarding the formation of the analogous Mo(IV)-nitrido complex lead to the conclusion that the mechanism of catalytic ammonia formation mediated by the title systems does not involve N-N cleavage of a dinuclear Mo-dinitrogen complex, but follows the classic Chatt cycle.

## 1. Introduction

Ammonia is one of the most important basic chemicals. It is produced on a large scale using the Haber–Bosch process and mainly used for the synthesis of fertilizers [1,2,3]. In nature, the enzyme nitrogenase binds dinitrogen and converts it into ammonia. This process takes place at the active center, the FeMoco [4,5,6,7,8,9,10,11]. In synthetic nitrogen fixation, model systems capable of converting dinitrogen into ammonia are developed, following the example of nitrogenase. The first dinitrogen complex was synthesized by Allen and Senoff in 1965 [12]. Chatt et al. established and elucidated the first mechanism of transition metal-mediated N_2_ reduction [13,14]. Since then, many other systems have been developed. The majority of catalytic systems are based on molybdenum and iron centers, which are also present in the FeMoco [15,16,17,18,19,20,21,22]. For the stepwise reduction and protonation of the dinitrogen ligand and the corresponding catalysis, respectively, acids such as [LutH]OTf (2,6-dimethylpyridine trifluoromethanesulfonate) or [ColH]OTf (2,4,6-trimethylpyridine trifluoromethansulfonate) as proton source and various metallocenes such as CoCp*_2_ or CrCp*_2_ as electron source have been used [18,19,20]. In 2019, Nishibayashi et al. introduced a new method for catalytic N_2_ fixation [23]. H_2_O is used as the proton source, coordinating to the [SmI_2_(thf)_2_] complex which donates the electrons, thus forming a PCET reagent. Employing this reagent instead of separate electron and proton donors led to a significant increase of the ammonia yield in catalytic experiments [23,24].

Based on their favorable properties in small-molecule activation, many different pincer ligands have been introduced over the last years [25,26,27,28,29,30,31]. PN^3^P pincer ligands containing amine groups between a central pyridine moiety and the terminal phosphine donors were developed by Schirmer et al. [27] and investigated by Kirchner et al. [28,29,32,33] with regard to their coordination to different metal centers, such as molybdenum. In this context, a range of catalytically active metal complexes were prepared [29,32]. Previously, these systems were also investigated in relation to their suitability for catalysts in synthetic nitrogen fixation [20]. Dinitrogen complexes, as far as they could be obtained, were also investigated and characterized [20]. In ref. [34], Nishibayashi and coworkers compared Mo(V)-nitrido complexes supported by the “classic” PNP pincer ligand to the Mo(V)-nitrido complex [Mo(N)Cl(H-PN^3^P^tBu^)]^+^ supported by a PN^3^P-ligand with respect to catalytic ammonia formation, using metallocenes (CoCp_2_, CrCp*_2_) as reductants and lutidinium triflate as acid. They found that the latter complex is catalytically inactive, in contrast to the systems supported by PNP ligand. In ref. [20], we showed that, using CrCp*_2_ as reductant and lutidinium triflate as acid, the complex [MoCl_3_(H-PN^3^P^tBu^)] is able to generate 3.1 equivalents of NH_3_ from N_2_. This suggests that the corresponding Mo-nitrido complex may be catalytic as well, in contrast to Nishibayashi’s initial result of ref. [34].

Due to the aforementioned increase in ammonia yield using SmI_2_/H_2_O, molybdenum complexes supported by PN^3^P-ligands herein are also examined in conjunction with this reagent in order to check whether a significant increase in ammonia yields is possible. Moreover, the title systems are also investigated regarding to the question of which mechanism applies to the N_2_-to-NH_3_ conversion. Currently, two scenarios are postulated for molybdenum complexes active in nitrogen fixation: firstly, Chatt’s distal mechanism [13,14] and secondly, the mechanism postulated by Nishibayashi et al. [35] for his pincer systems, which proceeds via a N-N cleavage of a bridged Mo(I)-dinitrogen complex. Finally, the substituents on the P-donors as well as on the amine groups in the ligand backbone are varied on order to investigate the influence of these changes on the catalytic activity of the derived complex.

## 2. Results and Discussion

**Scheme 1:**
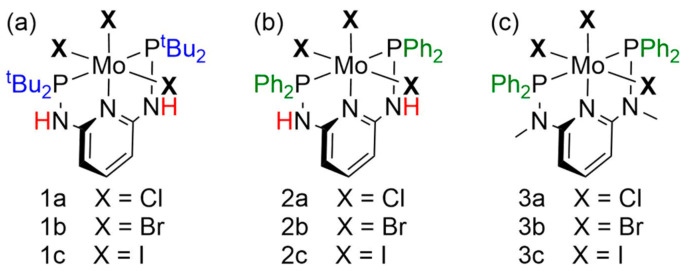
Complexes investigated in this study. (**a**) [MoX_3_(H-PN^3^P^tBu^)] (X = Cl, Br, I) complex **1a**–**c** has a *tert*-butyl phosphine group and a secondary amine-bridge to the pyridine. (**b**) [MoX_3_(H-PN^3^P^Ph^)] (X = Cl, Br, I) complex **2a**–**c** has a diphenylphosphino in place of the *tert*-butyl group and a secondary amine as a bridge. (**c**) [MoX_3_(Me-PN^3^P^Ph^)] (X = Cl, Br, I) complex **3a**–**c** has the same diphenylphosphino group but a tertiary amine with a methyl group as a bridge between the phosphine and the pyridine. The indices a, b, and c indicate which halogen is coordinated to the molybdenum, a = Cl, b = Br, c = I.

In our previous study, the three PN^3^P ligands H-PN^3^P^tBu^, H-PN^3^P^Ph^, and Me-PN^3^P^Ph^ were coordinated to MoX_3_ precursors (X = Cl, Br, I; see Scheme 1) and reduced to corresponding dinitrogen complexes in the presence of various monophosphines [20]. Furthermore, initial catalytic N_2_ reduction experiments were carried out with the PN^3^P-MoX_3_ complexes of Scheme 1, using [ColH]OTf or [LutH]OTf as the proton source and CrCp*_2_ or CoCp_2_ as the electron source. The best results (3.12 equiv. per catalyst) were obtained with catalyst **1a** when [LutH]OTf and CrCp*_2_ were employed [20]. More recently, Nishibayashi et al. achieved a new record for ammonia production with the PNP and PCP pincer systems using the PCET reagent SmI_2_ and water/ethylene glycol [23]. To determine if a similar increase in ammonia formation is possible with molybdenum PN^3^P pincer complexes, these are tested under similar conditions.

The different ligands were synthesized and coordinated to molybdenum(III) precursors to form the corresponding Mo(III) halido complexes **1a**–**c**, **2a**–**b**, **3a**–**b** (see Scheme 1), as described previously [20]. Complexes **2c** and **3c** are new and were synthesized in analogy to the others [20].

The catalysis was performed after the protocol introduced by Nishibayashi et al. [23]. Water and samarium iodide form the PCET reagent, serving as the proton and electron source. First, 180 equiv. of water and 180 eq. of SmI_2_(thf)_2_ were dissolved in THF and stirred for 10 min. Then, 1 equiv. of catalyst (**1a**–**c**, **2a**–**c**, **3a**–**c**, **4**) was diluted in THF and added to the mixture. The solution was stirred in a closed vessel under N_2_ atmosphere overnight until the blue solution decolorized. The resulting ammonia was transferred into 15 mL of a cold 2 M solution of HCl in ether using a constant N_2_ flow, and the formed ammonia was quantified by the Berthelot reaction [36]. The results are collected in Table 1.

Catalysts **2a**–**c** and **3a**–**c** (run 4**–**9) show no catalytic activity as they do not significantly exceed the stoichiometric limit of 2 equiv. (Table 1). Therefore, the change from the secondary amine to the tertiary amine has no significant influence on the activity of the catalyst. Notably, the dinitrogen complex derived from **2** (containing two monophosphine coligands) shows a less activated N_2_ ligand (ν_NN_ = 1962 cm^−1^) compared to the dinitrogen complex corresponding to **3** (1923 cm^−1^) [20]. Thus, it would have been expected that complexes **3a**–**c** have a significantly higher catalytic activity than their counterparts **2a**–**c**. Obviously, both systems are below a threshold required to mediate catalytic N_2_ reduction. In contrast, catalysts **1a**–**c** (run 1**–**3) are highly active, generating up to 39.7 equiv. of ammonia. Only minor changes in the ammonia yield were observed when changing the halide ligands. This observation also applies to the other systems [23,24], i.e., the halide ligands have no significant influence on the catalytic activity. The greatest influence thus is observed when going from the *tert*-butyl-phosphine group (**1a**–**c**) to the diphenylphosphino group (**2a**–**c**, **3a**–**c**).

**Scheme 2:**
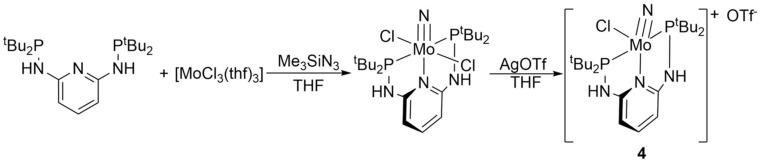
Synthesis of the molybdenum(V)-nitrido complex **4 [34]**.

Two mechanisms have been postulated for the catalytic conversion of N_2_ to NH_3_ mediated by pincer systems. In both mechanisms, the Mo(IV) nitrido complex **5** complex plays an essential role. The Mo(V) nitrido complex [MoCl(N)(H-PN^3^P^tBu^)](OTf) (**4**) was prepared first, as it is easier to isolate than its Mo(IV) counterpart **5** (see below). The synthesis of **4** (Scheme 2) was performed according to the literature with slight modifications [34].

**Figure 1:**
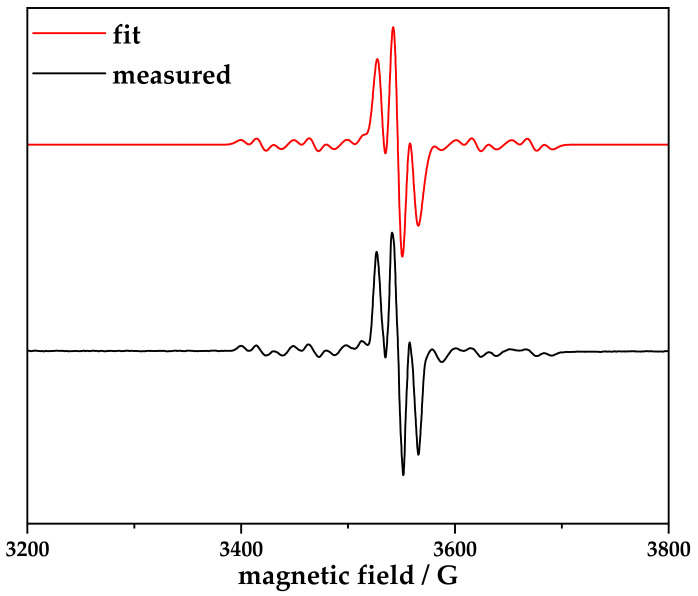
Measured liquid solution EPR spectrum (bottom, black) at 298 K in THF of complex **4**, with a natural abundance isotope mixture. The spectrum was measured at a microwave frequency 9.86 GHz and a power 0.2 mW using 100 mT field modulation at 100 kHz. Above in red is the fitted EPR spectrum shown.

Complex **4** was characterized using various spectroscopic and analytical methods. An X-ray crystal structure determination had been performed before, and a magnetic moment of µ_eff_ = 2.1 µ_B_ was determined by Evans–NMR spectroscopy [34]. In addition, we obtained an EPR spectrum of this complex (Figure 1, black) showing an S = 1/2 signal with additional hyperfine coupling to the nuclear spins of the I = 5/2 molybdenum isotopes ^95^Mo and ^97^Mo. Furthermore, a triplet splitting to the phosphorus nuclei can be observed. This splitting is evident for the main signal deriving from the I = 0 isotopes of Mo as well as for the hyperfine-spit signals. The coupling constants derived from a fit (red) are A(P) = 40 G and A(Mo) = 138.8 G. The isotropic g-value of the compound is 1.9862. Moreover, a HR-ESI mass spectrum as well as a ^31^P{^1^H} NMR spectrum (δ = 67.1 ppm) were obtained (Figures S1 and S5), being in agreement with the constitution of **4**. Due to the paramagnetic properties of **4,** the ^1^H NMR spectrum just shows broad signals and could not be analyzed.

Complex **4** was then investigated regarding its catalytic activity towards N_2_-reduction, using SmI_2_/H_2_O. It was found that, applying similar conditions as before (see above), it is able to generate 17 equiv. of ammonia. Thus, the complex is catalytically active, but does not reach the activity of the molybdenum(III) halogen systems. This may be due to various reasons. Firstly, the molybdenum center of **4** has an oxidation state of +V, whereas the nitrido intermediates in the postulated mechanisms are in the oxidation state +IV (see below). Due to the employed PCET reagent, the mechanism thus may run over “wrong” oxidation states, which could influence the catalytic activity. Moreover, catalytic experiments with Mo-pincer complexes were mostly performed with neutral species [20,23,24]; therefore, the positive charge of the complex could also have an influence on the yield of NH_3_. Finally, in the case of complex **4**, OTf^−^ has been introduced as counter anion. Previous studies have shown that the counter anion can greatly influence catalytic activity [37]. 

**Scheme 3:**
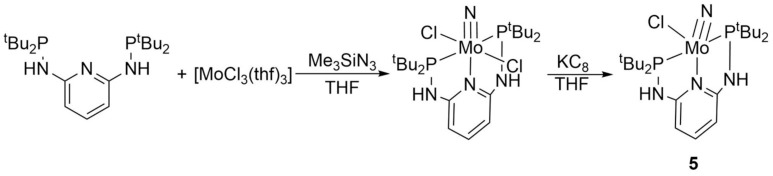
Reaction scheme for the synthesis of the molybdenum(IV) nitrido complex **5**.

Since the catalytic mechanism is thought to occur via a Mo(IV)-nitrido complex, the next goal was to synthesize this complex and investigate its catalytic activity. The synthesis was adapted from a protocol described by Nishibayashi et al. for the synthesis of Mo(IV)-nitrido complexes with PNP pincer ligands [38] (Scheme 3). [MoCl_3_(thf)_3_] was dissolved in THF and Me_3_SiN_3_ was added. The solution was stirred for 1 h at 50 °C. The solvent was removed and H-PN^3^P^tBu^, dissolved in THF, was added. The solution was stirred for 4 h at 50 °C. After cooling to room temperature, KC_8_ was added. The suspension was stirred overnight and then filtered over Celite. The solvent was removed, the residue was washed with hexane and dried under vacuum.

In contrast to **4**, chemical analysis and spectroscopic characterization of its one-electron reduced derivative **5** turned out to be difficult. A high-resolution ESI mass spectrum of **5** could be obtained (Figure S6). Notably, this is identical to that obtained for **4** since the monocation of **5** (being formed as a result of the ionization process) corresponds to the Mo(V)-nitrido complex **4**. The possibility that the mass spectrum derives from unreacted **4** can be excluded as it is very unlikely that a reduction with KC_8_ leaves the original Mo(V)-complex unaffected (see electrochemistry below). Thus we conclude that reduction of the Mo(V)- to the Mo(IV)-complex has in fact occurred, and the obtained ESI mass spectrum derives from the Mo(IV) product. This is supported by the fact that the Mo(V)-nitrido complex **4**, which previously had been visible in the NMR (see above, Scheme 2), could not be detected any more in the reaction product. Instead, the corresponding NMR spectra show a clean signal set that can be assigned to a compound with diamagnetic properties (Figures S2–S4). The signals agree with the NMR spectra of the free ligand H-PN^3^P^tBu^ regarding the chemical shifts and coupling constants. As the probability is very low that the signals of **5**, which are visible, are identical to those of the free ligand in all NMR spectra, we concluded that only the latter was detected in the solution. The results thus indicate that reduction of **4** has occurred, but the solid product **5** decomposes on a time scale of minutes upon re-dissolution.

**Figure 2:**
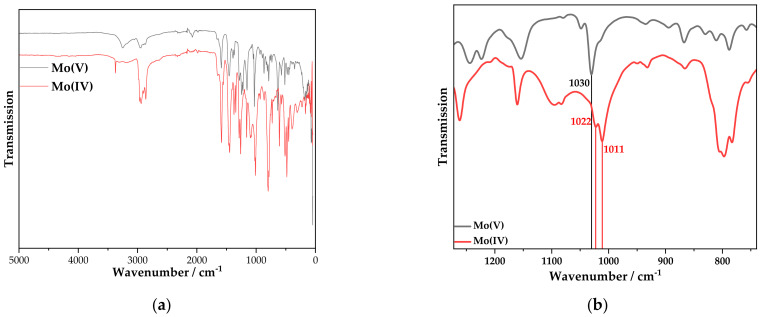
(**a**) IR spectra of the products **4** (black) and **5** (red). (**b**) Section of the IR spectra of the products **4** (black) and **5** (red) to highlight the Mo-N stretching vibration.

Further information on this issue is provided by vibrational spectroscopy. The IR spectrum of the solid product obtained from the synthesis of **5** (i.e., after evaporation of the solvent) is compared with the IR spectrum of **4** (Figure 2). Overall, the IR spectra (Figure 2a) show no major differences, which would be compatible with the fact that complexes **4** and **5** have the same structure except for the oxidation state of the Mo center. However, when comparing the two spectra in the 1000 cm^−1^ range, a shift of the Mo-N stretching vibration is visible (Figure 2b). Since complex **5** has a higher electron density at the Mo center than complex **4**, the triple bond between Mo and N is anticipated to be weaker in the former. Therefore, the band should shift to lower wavenumbers, in agreement with the observation. Specifically, the Mo(V)-nitrido complex **4** has a Mo-N stretching vibration band at 1030 cm^−1^. The Mo(IV)-nitrido complex **5** has two bands in this region, one (more intense) at 1022 and one (weaker) at 1011 cm^−1^. The fact that two bands appear in the Mo-N stretching region may be due to the fact that loss of the second chloride ligand upon one-electron reduction to the Mo(IV)-complex is incomplete, generating an admixture of a Mo(IV)-nitrido-dihalogenido species.

**Figure 3:**
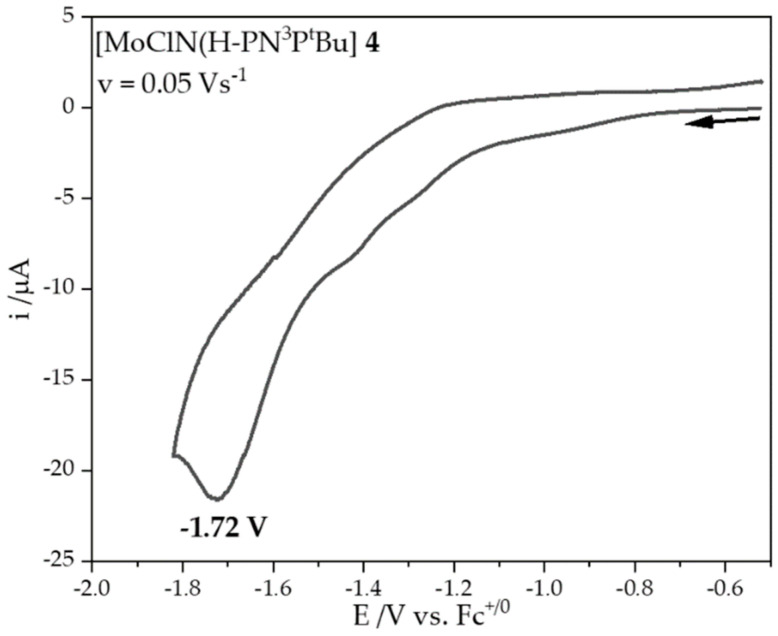
Cyclic voltammetry (E/V vs. Fc^+^/Fc) at a Pt working electrode (diam. 1 mm) of Mo(V)-nitrido **4** (1 mM) in THF/NBu_4_OTf (0.1 mM) at 0.05 Vs^−1^.

Cyclic voltammetry measurements were performed with complex **4** in order to determine the reduction potential of Mo(V) to Mo(IV) (Figure 3). In the corresponding CV of **4,** a reduction of Mo(V) to Mo(IV) occurs at −1.72 V vs. Fc^+^/Fc. Notably, this is far more negative than given by Nishibayashi et al. for this complex (−1.17 V vs. Fc/Fc+) [34] and slightly above the reduction potential of CoCp*_2_ (E_1/2_ = −1.85 V in THF) [39]. Therefore, this or any stronger reducing agent (e.g., KC_8_) should in fact allow a one electron reduction reaction. However, the reduction process is not reversible in the CV; i.e., re-oxidation of Mo(IV) to Mo(V) only occurs to a very small extent. Moreover, an increase of the scan rate did not lead to a reversible reduction and oxidation. To conclude, the Mo(IV)-nitrido complex **5** is probably present in the solid reaction product deriving from the reduction of **4**, but in solution it seems to decompose irreversibly. Optimization of the synthesis by changing the solvents, reaction times, and substitution of the halides led to the same results.

**Scheme 4:**
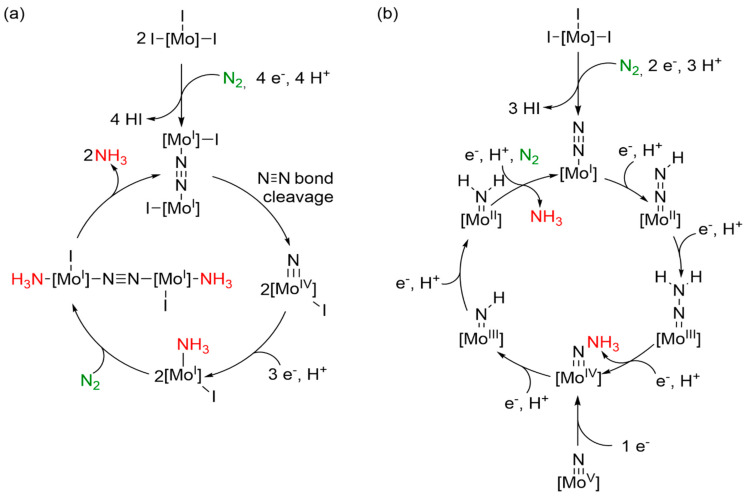
(**a**) Schematic representation of the postulated mechanisms from Nishibayashi et al. for pincer ligand systems [35]. (**b**) Schematic representation of a Chatt-type, distal mechanism with a PCET reagent [13,14,35,41].

Nishibayashi et al. postulated the pathway shown in Scheme 4a to account for the SmI_2_/H_2_O-mediated ammonia generation observed with their pincer systems [35,38,40]. Here, the molybdenum(III) halide complex is reduced to a dinitrogen-bridged dimolybdenum(I) complex. Subsequently, the N-N bond is spontaneously cleaved and two Mo(IV)-nitrido complexes are formed. The nitrido complexes are protonated and reduced three times, forming mononuclear Mo(I) ammine complexes. Afterwards, N_2_ coordinates, forming a dinitrogen-bridged diammine-complex. In the last step, ammonia decoordinates and the initial complex is obtained.

**Scheme 5:**
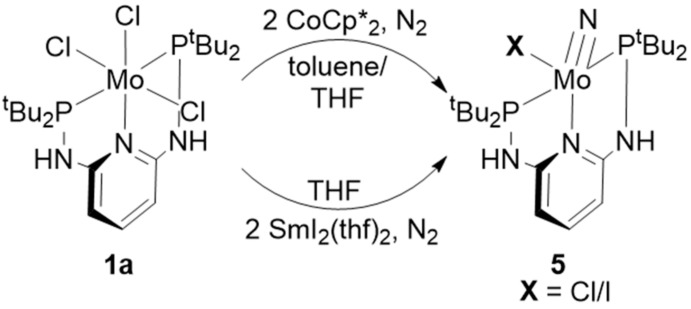
Reaction scheme for the N-N-cleveage experiments according to the literature [23,42]. Neither reaction afforded the Mo(IV)-nitrido complex **5**.

In order to evaluate this mechanism for our PN^3^P-pincer complex, N-N-cleavage experiments (Scheme 5) were carried out according to the literature [23,42]. To this end, the molybdenum(III) complex was dissolved in toluene or THF and reacted with 2 eq.s of CoCp*_2_ or 5 eq.s of SmI_2_(thf)_2_ under N_2_ for 15 min. The solvent was removed in vacuo and the residue was washed three times with pentane.

To identify the Mo(IV)-nitrido complex **5** as a cleavage product, high-resolution ESI mass spectrometry was employed instead of NMR spectroscopy, due to the low stability of this complex **5** in solution. However, **5** could not be detected in the mass spectrum as a product from these N-N cleavage experiments, in contrast to the reaction involving one-electron reduction of the Mo(V)-nitrido complex (see above, Scheme 2). In addition, changes in the experimental procedures, e.g., reaction time or equivalents, did not lead to positive results (Figures S7 and S8). Based on these findings, we conclude that the Mo(IV)-nitrido complex is not formed as a product of N-N cleavage. If the catalysis would proceed via N-N cleavage, the Mo(IV)-nitrido complex should have been obtained in the corresponding experiments. Since it could not be detected, the catalytic cycle does not involve N-N cleavage at the level of a dinuclear Mo(I) complex.

In an alternative, Chatt-type pathway (Scheme 4b) [13,14,35], the molybdenum(III) halide complex is reduced to a molybdenum dinitrogen complex which is protonated/reduced three times by SmI_2_/H_2_O, forming the first equivalent of ammonia and a molybdenum-nitrido complex. If a Mo(IV)-nitrido complex is present at this stage, the starting dinitrogen complex should be Mo(I)-N_2_. Formation of the Mo(IV)-nitrido complex is also possible by one-electron reduction of the Mo(V)-nitrido complex. The nitrido ligand of the former complex is also protonated and reduced three times. At the end of the cycle, ammonia is released and catalyst is regenerated. The major difference between the two mechanisms is the pathway leading to the Mo(IV)-nitrido complex. In the Chatt cycle (b), the distal nitrogen undergoes concerted protonation and reduction steps to reach the Mo(IV)-nitrido stage. In comparison, in the Nishibayashi mechanism (a), N-N cleavage occurs to form this species.

Coming back to the initial question regarding the influence of changes in the ligand system on the catalytic activity of the derived molybdenum complexes, it can be stated that only di-*tert*-butyl substituted P-donors seem to be able to confer the necessary activation to the N_2_-ligand of the parent dinitrogen complex to undergo protonation and reduction to ammonia. Diphenylphosphine groups, by contrast, are not capable of providing this activation, regardless of whether the amine function within the ligand backbone is methylated or not.

## 3. Materials and Methods

All reactions were performed under a nitrogen atmosphere using Schlenk techniques. The solvents were dried and freshly distilled under argon atmosphere prior to use. All starting materials were supplied from Sigma-Aldrich Co. and abcr GmbH & Co. KG and used as received. The ligands H-PN^3^P^tBu^ [28], H-PN^3^P^Ph^ [27], and Me-PN^3^P^Ph^ [32] were prepared according to the literature. [MoCl_3_(thf)_3_] [43], [MoBr_3_(thf)_3_] [44], and [MoI_3_(thf)_3_] [45] were prepared according to literature. The complexes **1a**–**c**, **2a**/**b** and **3a**/**b** were prepared according to literature [20].

Spectroscopic Characterization: IR spectra were obtained using a Bruker ALPHA-P-Spectrometer. FT-Raman spectra were recorded with an IFS 66/CS NIR Fourier-transform-Raman-spectrometer and a FRA 106 from Bruker (range from 3300 cm^−1^ to 20 cm^−1^ at a resolution of 20 cm^−1^). NMR spectra were recorded on a Bruker Avance III HD 400 pulse Fourier Transform spectrometer operating at a ^1^H frequency of 400.13 MHz and a ^31^P frequency of 161.98 MHz. Referencing was performed with tetramethylsilane (δ(^1^H) = 0 ppm) and 85% H_3_PO_4_ (δ(^31^P) = 0 ppm) serving as substitutive standards. Deuterated solvents for NMR measurements were purchased from Deutero and used as supplied.

Elemental analyses were performed with a EuroEA 3000 Elemental Analyzer. Samples were burned in sealed tin containers in a stream of oxygen.

High-resolution ESI mass spectra (HR-ESI) were measured with a Thermo Scientific Q Exactive Plus with a heated ESI unit.

Parallel Mode cw X-band EPR Spectra were collected using a Bruker EMXplus spectrometer with a PremiumX microwave bridge equipped with a dual mode cavity (Bruker ER-4116DM). EPR data collection was managed using the Bruker Xenon 1.0 software package. Spectral simulations were performed using the EPR simulation software package EasySpin **[46]**.

Electrochemical studies were performed with a home-made 3-electrode cell (WE: Pt, RE: Ag in a 1 mM THF/(NBu_4_)Otf solution, 0.1 M, CE: Ag). Ferrocene was added at the end of the experiments to determine the exact redox potential values. The potential of the cell was controlled by an EG&G 273A potentiostat.

[MoI_3_HPN^3^P^Ph^] 2c

The ligand H-PN^3^P^Ph^ (300 mg, 629 µmol) was dissolved in 20 mL toluene. The precursor [MoI_3_(thf)_3_] (359 mg, 569 µmol) was added to the solution. The suspension was stirred for 6 h under reflux. Afterwards the mixture was stirred for 14 h at rt. Next, the solid residue was filtered and washed with toluene, ether, *n*-hexane and THF.

Yield: 463 mg (486 µmol, 85%).

Anal. Calc. (%) for C_29_H_25_I_3_MoN_3_P_2_ (M = 954 g.mol^−1^): C, 36.5; H, 2.6; N, 4.4; found: C, 36.5; H, 3.0; N, 4.7.

IR (ATR): 3050, 2979, 2933, 2898, 1584, 1480, 1453, 1433 cm^−1^.

[MoI_3_MePN^3^P^Ph^] 3c

The ligand Me-PN^3^P^Ph^ (200 mg, 397 µmol) was dissolved in 10 mL toluene. The precursor [MoI_3_(thf)_3_] (262 mg, 378 µmol) was added to the solution. The suspension was stirred for 6 h under reflux. Afterwards the mixture was stirred for 2 d at 80 °C and 2 d at rt. Next, the solid residue was filtered and washed with toluene, ether, *n*-hexane, THF, and DCM.

Yield: 250 mg (286 µmol, 76%).

Anal. Calc. (%) for C_31_H_29_I_3_MoN_3_P_2_ (M = 582.2 g.mol^−1^): C, 37.9; H, 3.0; N, 4.3; found: C, 37.8; H, 3.2; N, 4.5.

IR (ATR): 3050, 2979, 2933, 2898, 1584, 1480, 1453, 1433 cm^−1^.

[Mo(V)ClN(H-PN^3^P^tBu^)]OTf 4

The complex was synthesized after Nishiabayashi et al. [38]; [MoCl_3_(thf)_3_] (211.8 mg, 0.51 mmol) and Me_3_SiN_3_ (70 µL, 0.53 mmol) were dissolved in 10 mL THF and stirred for 1 h at 50 °C. The solution was concentrated under reduced pressure and H-PN^3^P^tBu^ (203 mg, 0.51 mmol) in 10 mL THF was added via a syringe. The solution was stirred for 4 h at 50 °C. After concentrating under reduced pressure, the residue was washed 3x with *n*-hexane. AgOTf (83.5 mg, 0.32 mmol) suspended in 10 mL THF and added to the residue. The solution was stirred for 16 h at rt. After removing the solvent under reduced pressure, the residue was washed 3x with hexane and diluted again in THF. The suspension was filtered through Celite, the filter cake was washed 3x with 5 mL THF. The solution was concentrated under reduced pressure. After slowly adding benzol and *n*-hexane a light brown solid was obtained by filtration. The solid was dried under reduced pressure and stored under nitrogen atmosphere.

Yield: 180 mg (260 µmol, 81%).

MS: C_21_H_41_ClMoN_4_P_2_^+^ Calc.: *m*/*z* = 544.15437 found: *m*/*z* = 544.15413.

Anal. Calc. (%) for C_22_H_41_ClF_3_MoN_4_O_3_P_2_S (M = 692.01 g.mol^−1^): C, 38.2; H, 6.0; N, 8.1; S, 4.6 found: C, 35.1; H, 6.6; N, 8.0; S, 4.5. The high fluorine content falsifies the elemental analysis.

IR (ATR): 3253, 2944, 2870, 2073, 1588, 1455, 1389, 1283, 1242, 1221, 1156, 1030, 867, 786, 636, 571, 516 cm^−1^.

^31^P{^1^H} NMR: (THF-d_8_, 162.0 MHz, 300.0 K) δ = 67.1 (s, 2 P, P^t^Bu) ppm.

General procedure for catalytic experiments:

First, 3.6 mL of a SmI_2_(thf)_2_ solution (0.1 M, 360 µmol, 180 equiv.) in THF and 1.4 mL of H_2_O (0.26 M, 360 µmol, 180 equiv.) in THF were mixed for 5 min. In the meantime, 1 equiv. (2 µmol) of the catalyst (**1**–**3**) was dissolved in 2 mL THF. After adding the catalyst to the solution, it was stirred in a closed vessel under N_2_ atmosphere over night at rt. The color of the mixture thereby changed from blue to yellow. The generated ammonia in the solution was driven out with a stream of N_2_ through a glass bridge into a cold trap (cooled with acetone/N_2_) filled with 20 mL 2 M ethereal HCl solution. To drive off remaining ammonia, 20 mL of a 0.125 M solution of NaOH in methanol was added to the reaction solution. After expulsion of all NH_3_, the solvent from the cold trap was removed in vacuum. The resulting NH_4_Cl was quantified by the Berthelot reaction.

## 4. Conclusions

It could be shown that the use of SmI_2_(thf)_2_ and H_2_O leads to a significant increase of the ammonia yield in the catalysis mediated by Mo-PN^3^P complexes. However, the systems with phenyl-phosphine are still not catalytically active, only with *tert*-butyl-phosphine could catalytic activity be established. Unfortunately, samarium iodide and the synthesis of the catalyst are too expensive to be considered for large-scale application. To understand the mechanism of the catalytic cycle, the Mo(V)-nitrido complex **4** was first synthesized and characterized. It also exhibited a catalytic activity, generating 17 equiv. of ammonia. The Mo(V)-nitrido complex **4** could be reduced to the Mo(IV)-nitrido complex **5**, but detailed characterization of the latter species was hampered by its instability. Furthermore, N-N cleavage experiments were carried out. The Mo(IV)-nitrido complex **5** could not be detected in any of these experiments. Therefore, it is concluded that the catalytic cycle of the PN^3^P-systems follows a Chatt-type, distal mechanism of N_2_-reduction.

## Figures and Tables

**Table 1:** Results of catalysis with the PN^3^P-Mo(III) halide complexes with 180 equiv. SmI_2_/H_2_O.

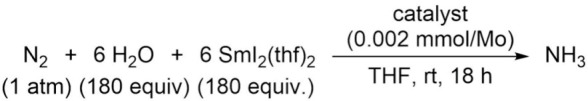			
Run	Catalyst	NH_3_/equiv.	NH_3_/%
1	**1a**	38.5 (±0.3)	64.2
2	**1b**	39.7 (±0.1)	66.2
3	**1c**	32.4 (±0.1)	54.0
4	**2a**	1.4 (±0.1)	2.3
5	**2b**	1.5 (±0.2)	2.5
6	**2c**	2.3 (±0.1)	3.8
7	**3a**	1.8 (±0.1)	3.0
8	**3b**	1.9 (±0.1)	3.3
9	**3c**	1.7 (±0.1)	2.8
10	**4**	17.0 (±1.0)	28.3

## Data Availability

Not applicable.

## Supplementary Materials

The following supporting information can be downloaded at: https://www.mdpi.com/article/10.3390/molecules27227843/s1, Figure S1: ^31^P{^1^H} NMR Spectrum of Mo(V)-nitrido complex **4** in THF-d_8_. Impurities are caused by slow decomposition in solution. Figure S2: (a) ^31^P{^1^H} NMR Spectrum of Mo(IV)-nitrido **5** in THF-d_8_. (b) ^31^P{^1^H} NMR Spectrum of H-PN^3^P^tBu^ in THF-d_8_. Figure S3: (a) ^1^H NMR Spectrum of Mo(IV)-nitrido **5** in THF-d_8_. (b) ^1^H NMR Spectrum of H-PN^3^P^tBu^ in THF-d_8_. * Signals of the Ligand H-PN^3^P^tBu^, # Educt from the synthesis of the ligand H-PN^3^P^tBu^. Figure S4: (a) ^13^C{^1^H} NMR spectrum of Mo(IV)-nitrido complex **5** in THF-d_8_. (b) ^13^C{^1^H} NMR Spectrum of H-PN^3^P^tBu^ in THF-d_8_. * Signals of the Ligand H-PN^3^P^tBu^, # Educt from the synthesis of the ligand H-PN^3^P^tBu^. Figure S5: HR-ESI mass spectrum of the Mo(V)-nitrido **4**. The measured spectrum is shown in black (top) and the simulated spectrum is shown in green (bottom), when the signal positions match. Figure S6: HR-ESI mass spectrum of the Mo(IV)- nitrido complex **5**. The measured spectrum is shown in black (top) and the simulated spectrum is shown in green (bottom), when the signal positions match. Figure S7: HR-ESI mass spectrum of the N-N-cleavage experiment with [MoCl_3_(H-PN^3^P^tBu^)] and CoCp*_2_ in THF. The measured spectrum is shown on the top and the simulated spectrum is shown below. The signals do not fit. Figure S8: HR-ESI mass spectrum of the N-N-cleavage experiment with [MoCl_3_(H-PN^3^P^tBu^)] and CoCp*_2_ in toluene. The measured spectrum is shown on the top and the simulated spectrum is shown below (red and green). The signals marked in green of the simulated spectrum agree with the measured spectrum. The red signals in the simulated cannot be found in the measured spectrum. Overall, the signals do not fit. Figure S9: HR-ESI mass spectrum of the N-N-cleavage experiment with [MoCl_3_(H-PN^3^P^tBu^)] and SmI_2_(thf)_2_ in THF. The measured spectrum is shown on the top. In the N-N cleavage experiment with SmI_2_(thf)_2_ Nishibayashi et al. only obtained the Mo(IV)-iodido nitrido complex [23]. Therefore the simulated spectrum of the analogous species is shown at the bottom (black). The signals do not fit.

## Sample Availability

Samples of the compounds are not available from the authors.
