# Supplementary material for: Catalytic Ammonia Synthesis Mediated by Molybdenum Complexes with PN3P Pincer Ligands: Influence of P/N Substituents and Molecular Mechanism
Source: Molecules. 2022 Nov 14;27(22):7843. doi: 10.3390/molecules27227843 (PMC9692791; doi:10.3390/molecules27227843)
Supplement: Supplementary file 1 [file molecules-27-07843-s001.zip › molecules-1929779-supplementary.pdf]

Supporting Information

Catalytic Ammonia Synthesis Mediated by Molybdenum Complexes with PN3P Pincer Ligands: Influence of P/N Substituents and Molecular Mechanism

Katja Bedbur,[a] Nadja Stucke,[a] Lina Liers,[a] Jan Krahmer[a] und Felix Tuczek[a]

[a] Institute for Inorganic Chemistry, Christian Albrechts University Kiel

E-Mail: ftuczek@ac.uni-kiel.de

Table of contents:

1. NMR Spectra
2. HR-ESI Spectra
3. References

1. NMR Spectra:

1.1 $^{31}P\{^1H\}$ NMR spectrum:

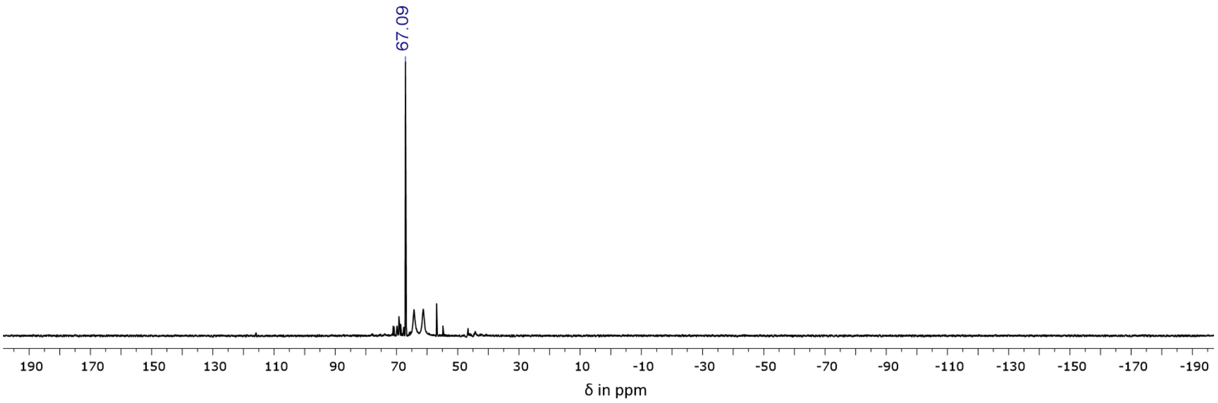

Figure S1. $^{31}P\{^1H\}$ NMR Spectrum of Mo(V)-nitrido complex **4** in THF-d_8. Impurities are caused by slow decomposition in solution.

1.2 NMR spectra of Mo(IV)-nitrido complex 5 compared with the free Ligand H-PN^{3}P^{t}Bu

1.2.1. $^{31}P\{^1H\}$ NMR spectra:

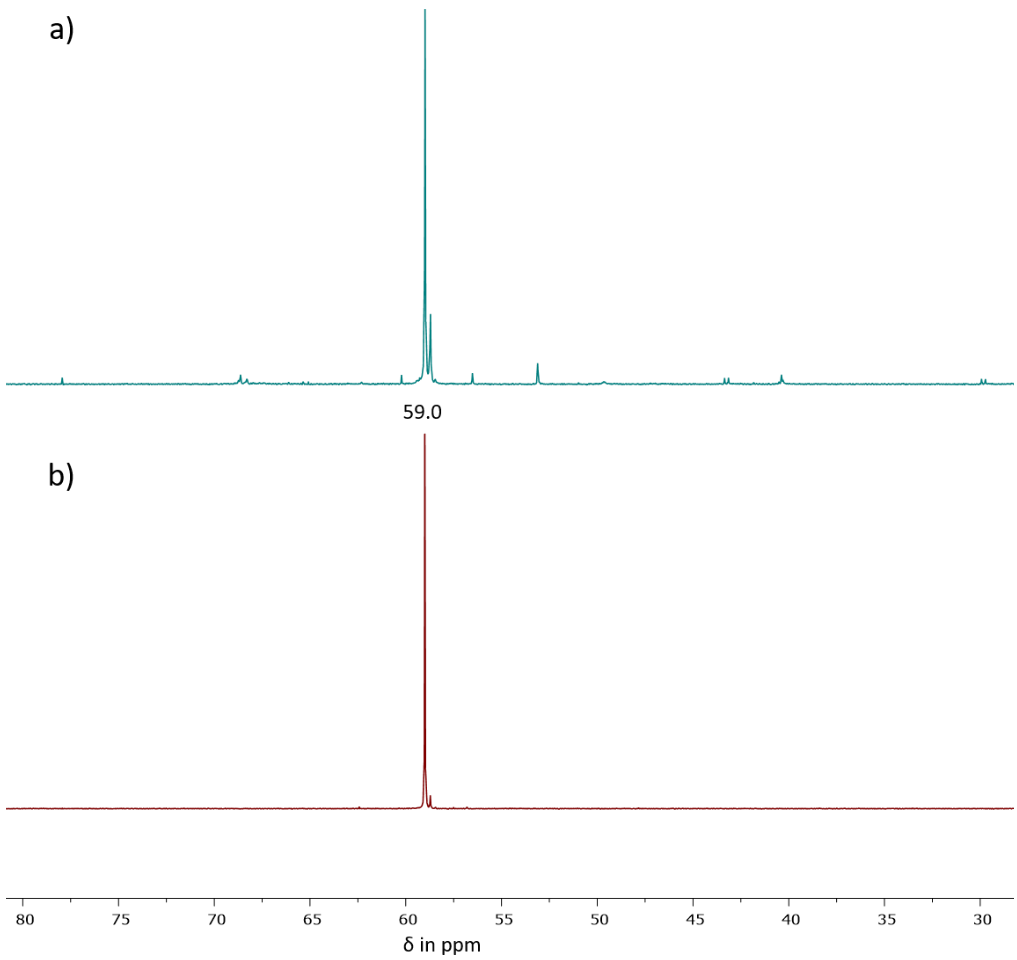

Figure S2. a) $^{31}P\{^1H\}$ NMR Spectrum of Mo(IV)-nitrido **5** in THF-d_8. b) $^{31}P\{^1H\}$ NMR Spectrum of H-PN^{3}P^{tBu} in THF-d_8.

1.2.2. ^{1}H NMR spectra:

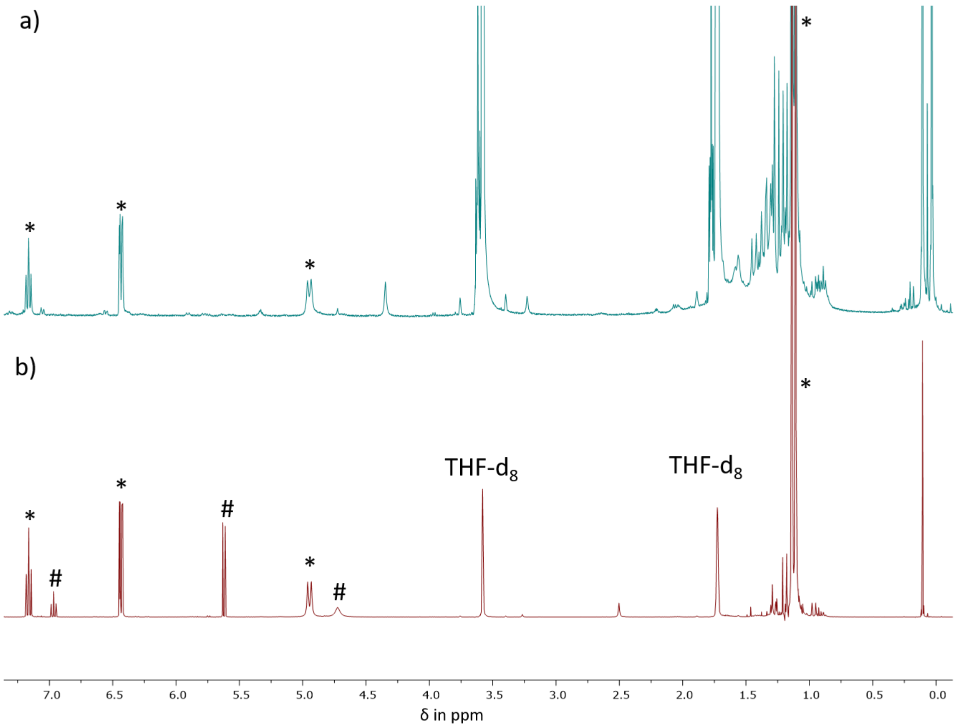

Figure S3. a) ^{1}H NMR Spectrum of Mo(IV)-nitrido **5** in THF-d_8. b) ^{1}H NMR Spectrum of H-PN^{3}P^{tBu} in THF-d_8. * Signals of the Ligand H-PN^{3}P^{t}Bu, # Educt from the synthesis of the ligand H-PN^{3}P^{tBu}.

1.2.3. ^{13}C{^{1}H} NMR spectra:

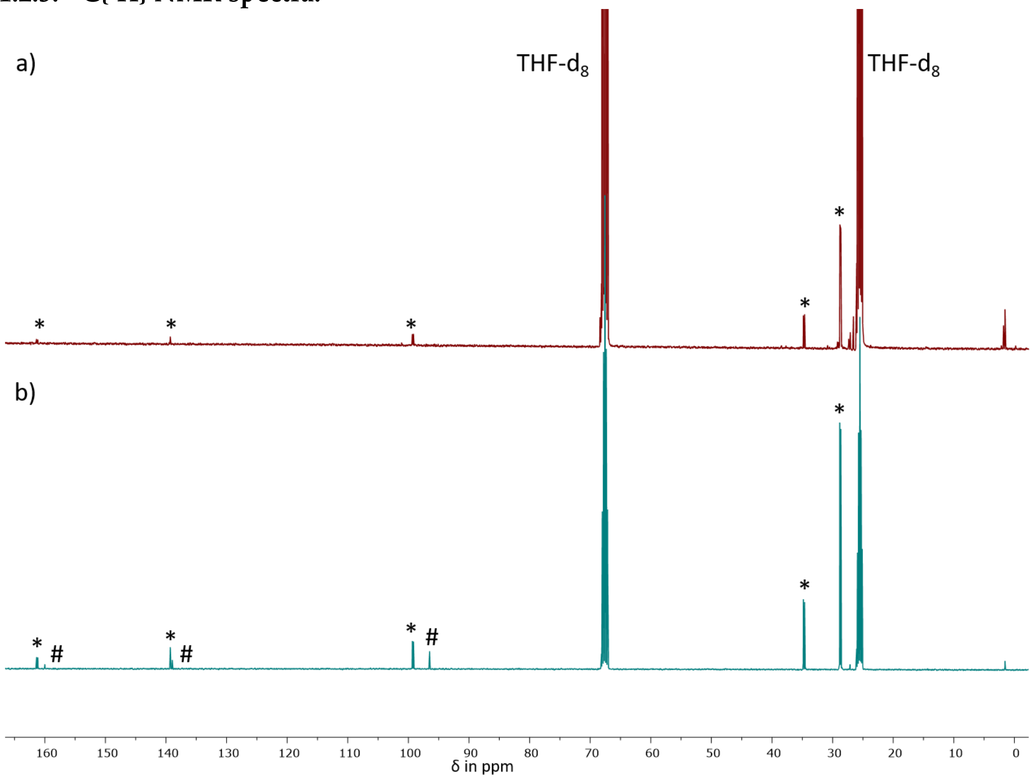

Figure S4. a) ^{13}C{^{1}H} NMR spectrum of Mo(IV)-nitrido complex **5** in THF-d_8. b) ^{13}C{^{1}H} NMR Spectrum of H-PN^{3}P^{tBu} in THF-d_8. * Signals of the Ligand H-PN^{3}P^{tBu}, # Educt from the synthesis of the ligand H-PN^{3}P^{tBu}.

2. HR-ESI spectra

2.1. HR-ESI spectrum of Mo(V)-nitrido complex 4:

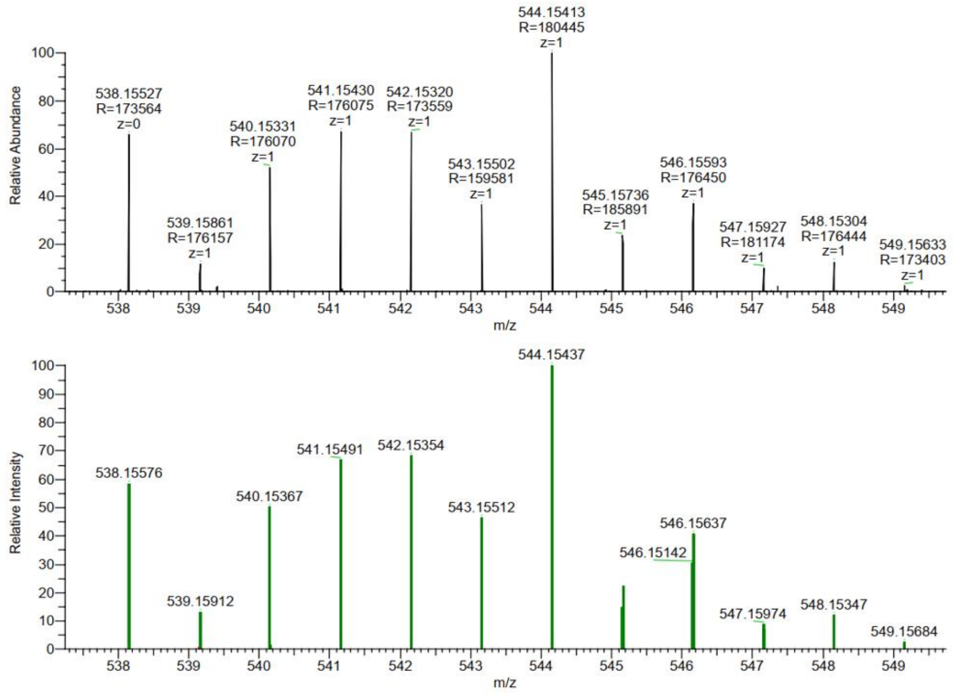

Figure S5. HR-ESI mass spectrum of the Mo(V)-nitrido **4**. The measured spectrum is shown in black (top) and the simulated spectrum is shown in green (bottom), when the signal positions match.

2.2. HR-ESI spectrum of Mo(IV)-nitrido complex 5:

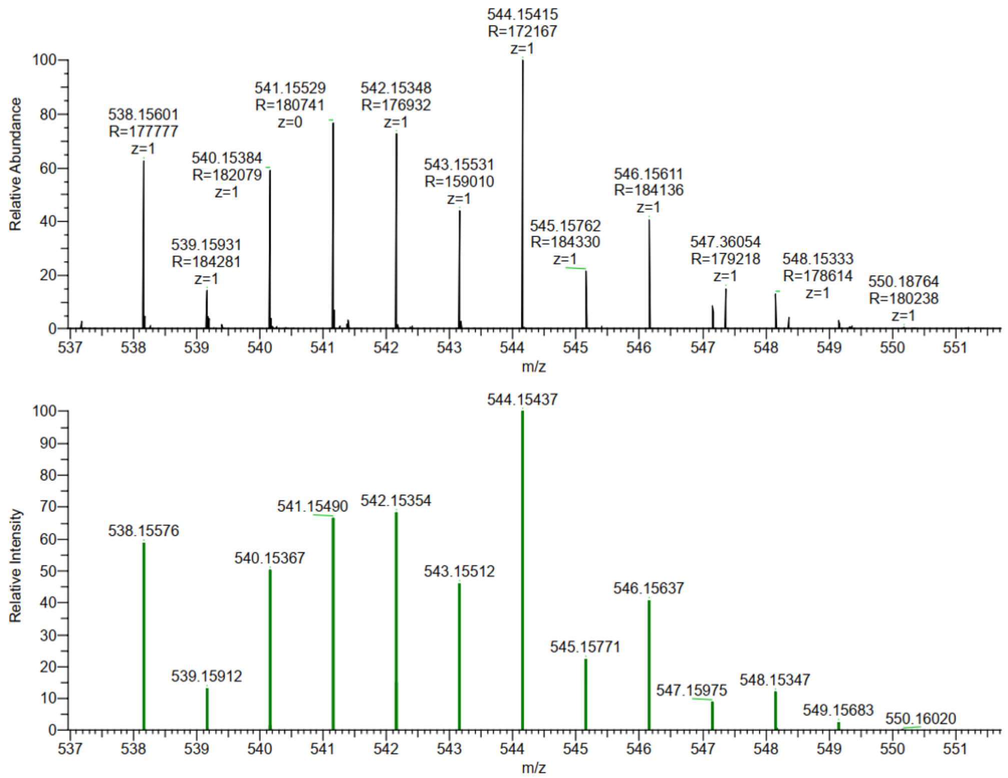

Figure S6. HR-ESI mass spectrum of the Mo(IV)- nitrido complex **5**. The measured spectrum is shown in black (top) and the simulated spectrum is shown in green (bottom), when the signal positions match.

2.3 HR-ESI spectra of the N-N-cleavage experiments:

2.3.1 HR-ESI spectrum of the N-N-cleavage experiment with [$MoCl_3$(H-PN^3P^{tBu})] and $CoCp^*_2$ in THF

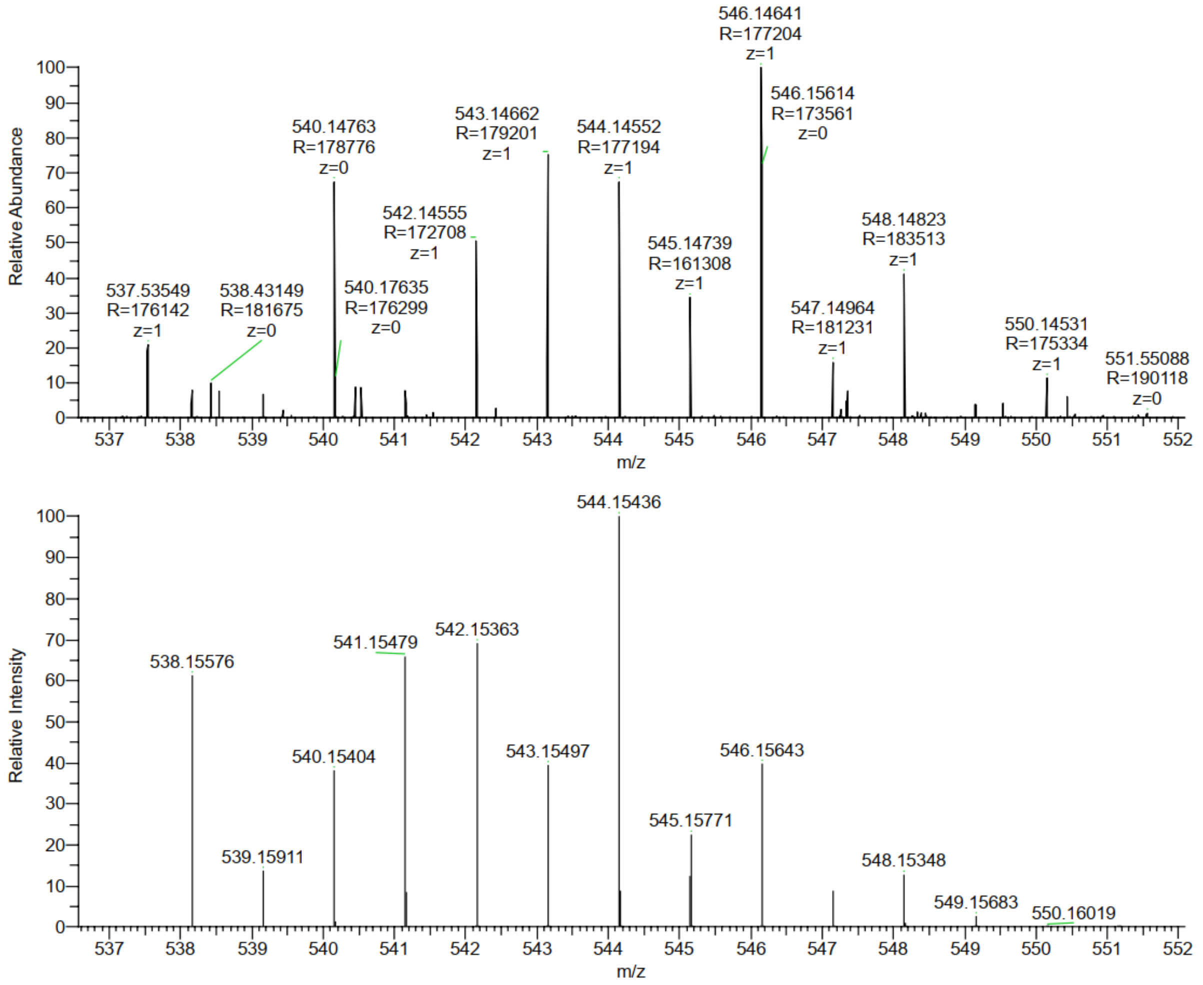

Figure S7. HR-ESI mass spectrum of the N-N-cleavage experiment with [$MoCl_3$(H-PN^3P^{tBu})] and $CoCp^*_2$ in THF. The measured spectrum is shown on the top and the simulated spectrum is shown below. The signals do not fit.

2.3.2 HR-ESI spectrum of the N-N-cleavage experiment with [MoCl3(H-PN3PtBu)] and CoCp*2 in toluene

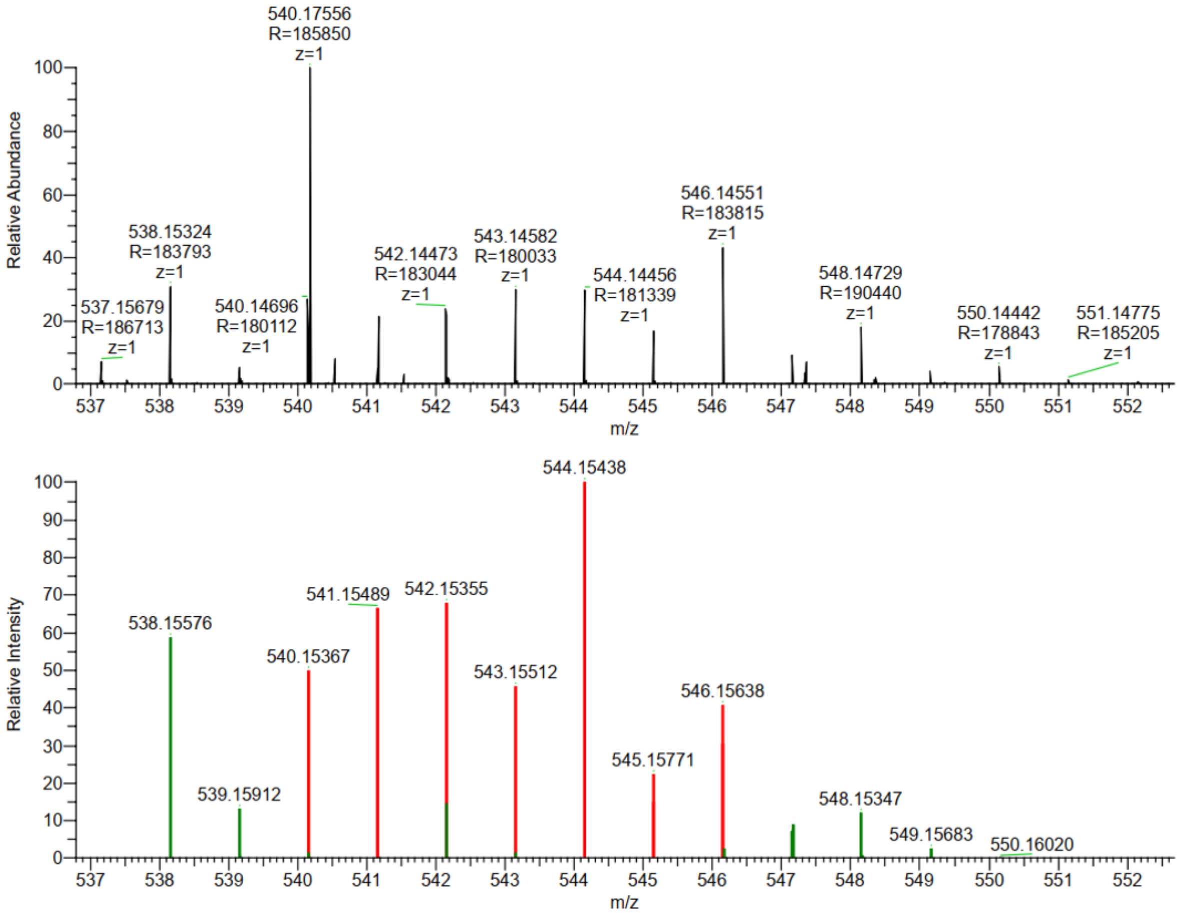

Figure S8. HR-ESI mass spectrum of the N-N-cleavage experiment with [MoCl3(H-PN3PtBu)] and CoCp*2 in toluene. The measured spectrum is shown on the top and the simulated spectrum is shown below (red and green). The signals marked in green of the simulated spectrum agree with the measured spectrum. The red signals in the simulated cannot be found in the measured spectrum. Overall, the signals do not fit.

2.3.3 HR-ESI spectrum of the N-N-cleavage experiment with $[MoCl_3(H\text{-}PN^3P^{tBu})]$ and $[SmI_2(thf)_2]$ in THF

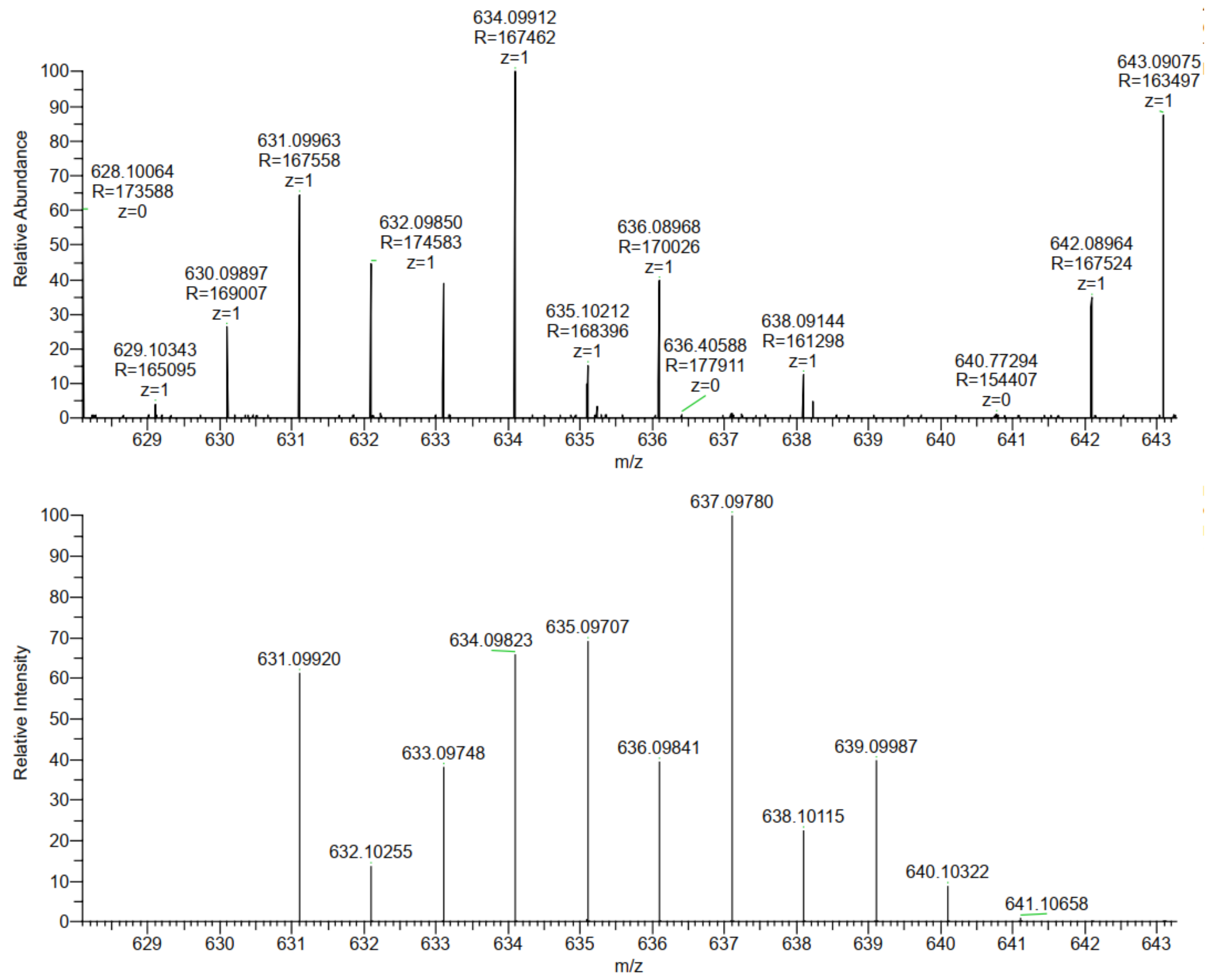

Figure S9. HR-ESI mass spectrum of the N-N-cleavage experiment with $[MoCl_3(H\text{-}PN^3P^{tBu})]$ and $SmI_2(thf)_2$ in THF. The measured spectrum is shown on the top. In the N-N cleavage experiment with $SmI_2(thf)_2$ Nishibayashi *et al.* only obtained the Mo(IV)-iodido nitrido complex [1]. Therefore the simulated spectrum of the analogous species is shown at the bottom (black). The signals do not fit.

3. References

S1. Ashida, Y.; Arashiba, K.; Nakajima, K.; Nishibayashi, Y. Molybdenum-catalysed ammonia production with samarium diiodide and alcohols or water. *Nature* **2019**, *568*, 536–540, doi:10.1038/s41586-019-1134-2.
